# Molecular Characterization of Two Known SRD5A2 Gene Variants in Mexican Patients With Disorder of Sexual Development

**DOI:** 10.3389/fgene.2021.794476

**Published:** 2022-01-27

**Authors:** Ortiz-López María Guadalupe, Sánchez-Pozos Katy, Aguirre-Alvarado Charmina, Pirkko Vihko, Menjivar Marta

**Affiliations:** ^1^ Laboratorio de Endocrinología Molecular, Research Division, Hospital Juárez de México, Mexico City, Mexico; ^2^ Laboratorio de Bioquímica Farmacológica, Departamento de Bioquímica, Escuela Nacional de Ciencias Biológicas, Instituto Politécnico Nacional, Mexico City, Mexico; ^3^ Unidad de Investigación Médica en Inmunología e Infectología, Centro Médico Nacional, La Raza, IMSS, Mexico City, Mexico; ^4^ Department of Clinical Chemistry and Hematology, University of Helsinki, Helsinki, Finland; ^5^ Laboratorio de Diabetes, Departamento de Biología, Universidad Nacional Autónoma de México, Mexico City, Mexico

**Keywords:** testosterone, 5-alpha-reductase deficiency, dihydrotestosterone, undefined genitalia, 46.XY

## Abstract

**Background:** The 5α-reductase type 2 deficiency (5α-RD2) is a specific form of disorder of sexual development (DSD). Pathogenic variants in the *SRD5A2* gene, which encodes this enzyme, are responsible for 46,XY DSD.

**Objective:** The objective of the study was to investigate the genetic etiology of 46,XY DSD in two Mexican families with affected children.

**Materials and methods:** The *SRD5A2* gene of the parents and affected children was screened in both families *via* polymerase chain reaction amplification and DNA direct sequencing. The role of genetic variants in enzymatic activity was tested by site-directed mutagenesis.

**Results:** Subject 1 presented two variants: p.Glu197Asp and p.Pro212Arg. Subject 2 was homozygous for the variant p.Glu197Asp. The two variants were reported in early studies. The directed mutagenesis study showed that the p.Glu197Asp and p.Pro212Arg variants lead to a total loss of enzymatic activity and, consequently, abnormal genitalia development in the patients.

**Conclusion:** These results suggest that p.Glu197Asp and p.Pro212Arg are pathogenic variants that lead to the phenotypic expression of DSD. 5α-RD2 is of extreme importance not only because of its frequency (it is rare) but also because of its significance in understanding the mechanism of androgen action, the process of sexual differentiation, and the factors that influence normal sexual behavior.

## 1 Introduction

Disorders of sex development (DSD) comprise a varied group of rare inherited conditions characterized by inconsistency among the anatomical, gonadal, and genetic sex (Lee et al., 2006). The etiological categories of non-syndromic 46, XY DSD include gonadal dysgenesis, defects in androgen biosynthesis, insensitivity to androgen action, and defects in the synthesis or action of anti-Müllerian hormone (Wisniewski et al., 2019).

The 5-alpha reductase type 2 deficiency (5α‐RD2) is a rare cause of undefined genitalia in male children (Acién and Acién, 2020). The differential diagnosis of DSD due to 5α‐RD2 is complex because other forms of DSD overlap with alternate male DSD, such as androgen insensitivity syndrome (partial form) and testosterone synthesis defects. Hence, an integral diagnosis is recommended, which involves clinical, hormonal, and molecular assessments.

In 1974, 5α‐RD2 was first described clinically and biochemically in studies of Dominican Republic subjects and in two siblings from Dallas, TX, United States (Imperato-McGinley et al., 1974; Walsh et al., 1974). 5α‐RD2 is an autosomal recessive 46,XY DSD caused by genetic variants in the SRD5A2 gene, manifested by variable degrees of undervirilization (Fan et al., 2020). The 5α‐RD2 manifests as failed or partial masculinization of external genitalia with normal Wolffian ducts, due to impaired activity of the 5-alpha reductase type 2 enzyme (3-oxo-5-alpha-steroid 4 dehydrogenase, 5α‐R2). The enzyme catalyzes the conversion of testosterone into dihydrotestosterone (DHT), which is essential for the normal *in utero* development of the male external genitalia, urethra, prostate gland, penis, and scrotum in the male fetus and plays a role in pubertal virilization (Kang et al., 2014). The gene *SRD5A2*, which encodes the enzyme 5α‐R2, is located on the band p23 on chromosome 2. The gene comprises five exons and encodes a 254 amino acid protein (28.4 kDa) (Wigley et al., 1994).

Individuals with 5α‐RD2, homozygous or heterozygous for loss of function variants in the *SRD5A2* gene, typically virilize strongly at puberty with the growth of the prostate and facial, pubic, and body hair, as well as enhanced activity of 5α‐R1, and residual activity of the mutated 5α‐R2 enzyme (Maimoun et al., 2011). In view of the anticipated development of secondary sexual characteristics in puberty, affected individuals with a correct diagnosis in infancy are usually advised a male sex assignment; however, in most cases, sex assignment is female (Hughes et al., 2006). Therefore, misdiagnoses have a substantial impact on physical, psychological, and social aspects (Özbey and Etker, 2013). Hence, an accurate diagnosis would allow adequate clinical and social management of affected patients.

Approximately 124 pathogenic variants have been described in the *SRD5A2* gene (Human Gene Mutation Database, http://www.hgmd.cf.ac.uk/ac/index.php). Avendaño et al. suggested that pathogenic variant positions in the *SRD5A2* gene may influence phenotype (Avendaño et al., 2018). Thus, in this study, we describe the clinical and molecular characteristics of two affected subjects from two states in Mexico.

## 2 Material and Methods

### 2.1 Subjects

#### 2.1.1 Subject 1

**FIGURE 1:**
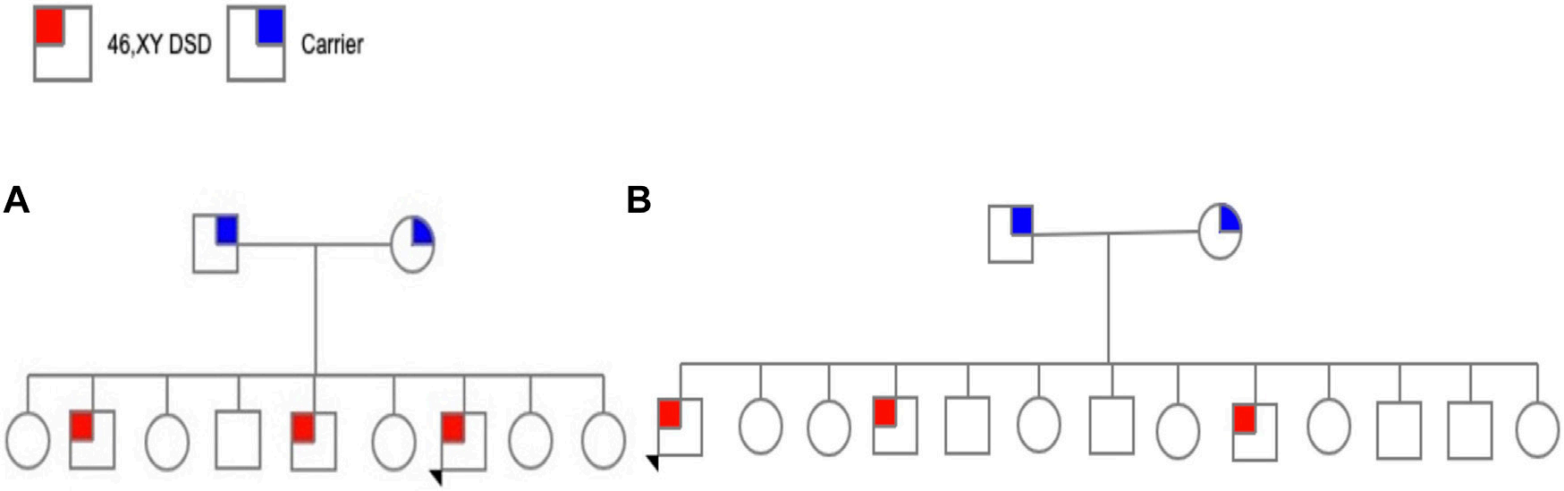
**(A)** Pedigree family 1 and **(B)** Pedigree family 2.

This subject was from a family from, Altotonga, Veracruz. The family comprises five daughters, one healthy man, and three affected individuals (aged 20, 18, and 8). Subject 1 was 8 years old. He was enrolled after obtaining voluntary informed consent from his parents and assent from the participant who was younger than 18 years old. He was referred for molecular studies because of manifestations of genital ambiguity and laboratory features indicative of 5α‐RD2 (Figure 1A).

#### 2.1.2 Subject 2

This subject was 50 years old, from Santa Clara, Hidalgo. He was one of 13 children (six daughters and seven sons), of which three were affected (aged 50, 40, and 28 years) and raised as females (Figure 1B).

Notably, there was no known consanguinity or history of the disease in the families of the two subjects.

The research protocol was approved by the ethics committee of Hospital Juárez de México (HJM-225/96), and written informed consent was obtained from all patients or legal guardians.

### 2.2 Clinical Characterization

The diagnosis of 5α‐RD2 was established in all patients by physical examination, measurements of plasma testosterone (T) and DHT, the T/DHT ratio, and the presence of a 46,XY karyotype (Ahmed et al., 2016).

### 2.3 Hormonal Assay

Total T measurements and DHT were performed using commercial radioimmunoassay kits (Diagnostic Products Corporation, Los Angeles, CA, United States), according to the instructions of the manufacturer. Testosterone and DHT levels were measured before and 48 h after three intramuscular injections of human chorionic gonadotropin (hCG) on alternate days using a 1,000 IU/3 days (sampling at days 0, 3, and 5) (Grant et al., 1976; Ahmed et al., 2016). The T/DHT ratio after hCG stimulation was also determined.

### 2.4 Molecular Analysis of SRD5A2 Gene

A peripheral blood sample (5 ml) was collected from all participants in EDTA tubes and stored on ice during transportation to the laboratory. Genomic DNA was extracted from the total blood samples, according to Miller et al. (1988). The DNA obtained was measured using a spectrophotometer, and the integrity was confirmed by 1% agarose electrophoresis. The DNA samples were stored at −20°C until analysis.

#### 2.4.1 Polymerase Chain Reaction and Sequencing

Coding sequence differences in the *SRD5A2* gene were identified by exon-specific polymerase chain reaction (PCR) and sequencing analysis. Exons 1–5 of the *SRD5A2* gene were amplified by PCR using pairs of specific primers for each exon (Table 1); the design primers are from Labrie et al. (1992). The cycling conditions were as follows: 5 min denaturation at 95°C, then 25 cycles of 94°C for 1 min, 54°C–65°C for 1 min, and 72°C for 1 min. The final cycle was 72°C for 5 min. PCR products were analyzed to verify the correct size fragments by using 1% agarose electrophoresis.

**TABLE 1:** Oligonucleotides used for amplification of each exon of the *SRD5A2* gene.

	Primer name	Sequence (5′ → 3′)	PCR fragments length (bp)	Annealing temperature (°C)
Exon 1	501	5′-GCA​GCG​GCC​ACC​GGC​GAG​G-3′	358	65
	502	5′-AGC​AGG​GCA​GTG​CGC​TGC​ACT-3′		
Exon 2	503	5′-TGA​ATC​CTA​ACC​TTT​CCT​CCC-3′	235	58
	504	5′-AGC​TGG​GAA​GTA​GGT​GAG​AA-3′		
Exon 3	505	5′-TGT​GAA​AAA​GCA​CCA​CAA​TCT-3′	208	58
	506	5′-CAG​GGA​AGA​GTG​AGA​GTC​TGG-3′		
Exon 4	507	5′-TGA​TTG​ACC​TTC​CGA​TTC​TT-3′	232	54
	508	5′-TGG​AGA​AGA​AGA​AAG​CTA​CGT-3′		
Exon 5	509	5′-TCA​GCC​ACT​GCT​CCA​TTA​TAT-3′	166	58
	510	5′-CAG​TTT​TCA​TCA​GCA​TTG​TGG-3′		

PCR products were sequenced using AmpliCycle Sequencing (Perkin Elmer, USA) after being purified by DNA columns (Centricon-100 columns; Amicon Inc., Beverly, MA, USA). The resulting sequences obtained from patients, controls, and parents were compared with the NCBI human reference sequence of the *SRD5A2* gene (European Molecular Biology Laboratory, EMBL; GenBank; and DNA Data Bank of Japan, DDBJ; nucleotide sequence databases under the accession number M74047).

#### 2.4.2 Direct Mutagenesis of the SRD5A2 Gene

The role of genetic variation in enzymatic activity was evaluated by directed mutagenesis using the human embryonic kidney 293 (HEK293) cell line (American Type Culture Collection, CRL 1573). cDNA was transfected into HEK293 cells.

A commercial expression vector containing the human full-length *SRD5A2* cDNA [GenBank NC_000002.12 (NM_000348.4)] pCMV6-XL1 (Invitrogen) was used as the template for p.Glu197Asp and p.Pro212Arg mutant construction. The mutants were constructed using the QuikChange™ Site-Directed Mutagenesis Kit (Stratagene Ltd., Cambridge, UK) and highly purified primers, according to the instructions of the manufacturer. The mutagenic oligonucleotide primers used were 197DF (5′-AAT​TTC​CTC​GGT​GAT​ATC​ATT​GAA​TGG-3′), 197D (5′-ATT​CAA​TGA​TAT​CAC​CGA​GGA​AAT​TGG​C-3′), and 212F (5′-TTG​GTC​CCT​CCG​AGC​ACT​TGC​ATT​TGC-3′), and 212R (5′-GCA​AGT​GCT​CGG​AGG​GAC​CAA​GTG​G-3′). High-quality wild-type and mutant plasmid cDNAs were isolated and purified using the QIAfilter Plasmid Midi and QIAfilter Plasmid Maxi Protocol (Qiagen, GmbH, Germany), according to the protocols of the manufacturer. SalI and NotI restriction enzyme sites were used to digest the *SRD5A2* cDNA, and the digests were analyzed *via* electrophoresis on 1% agarose gels. The mutated products were transformed into DH5α-T1 competent cells. Positive clones were picked and sequenced to corroborate the site-directed genetic variations. The nucleotide sequences of the wild-type constructs were verified by DNA sequencing (AmpliCycle Sequencing, Perkin Elmer, Q). All full-length mutated pCMV6-XL1 cDNAs were confirmed by DNA sequencing in both the sense and antisense strands to validate site-specific mutagenesis and to confirm that there were no off-target substitutions.

#### 2.4.3 Transfections and 5a-Reductase Assays

HEK-293 cells were preserved in Dulbecco’s modified Eagle’s medium supplemented with 10% stripped fetal calf serum, 100 U/ml penicillin, and 100 μg/ml streptomycin. Transfections were conducted in subconfluent HEK cells (2.9 × 10^6^ cells/plate), which were provided with 5% CO_2_ at 37°C. Wild-type and mutant plasmids were transfected into the cells at a concentration of 2.0 μg per plate by using the DOTAP Liposomal Transfection Reagent (Boehringer Mannheim) according to the instructions of the manufacturer. The cells were rinsed with phosphate-buffered saline (PBS), 48 h later, collected, and processed for enzymatic assays. 5α-R2 activity was determined in cell extracts in 0.1 M Tris-citrate buffers at the indicated pH with 1 nM ^14^C-testosterone (120 days p.m. pmol^−1^) as the substrate and 10 mM NADPH as the cofactor and increased concentrations of testosterone (0.125–8.0 μmol/L) (Andersson et al., 1991). Measurement of 5α‐R2 activity was performed in triplicate, and the activities were measured by the percentage of DHT detected by HPLC; results were represented as the percent of conversion to DHT (Dallob et al., 1994).

#### 2.4.4 Prediction of Functional Effects of Mutants SRD5A2

The functional prediction of substitutions in the human *SRD5A2* gene was analyzed using the following programs: PolyPhen2 (Polymorphism Phenotyping; http://genetics.bwh.harvard.edu/pph2/), PROVEAN (Protein Variation Effect Analyser; http://provean.jcvi.org/seq_submit.php), SIFT (Sorting Intolerant from Tolerant amino acid substitutions; http://sift.jcvi.org/www/SIFT_enst_ submit.html), and MUpro (predictions of protein stability changes upon genetic variations; http://mupro.proteomics.ics.uci.edu/).

#### 2.4.5 Structure Modeling

The polymorphic human SRD5A2 model was generated by homology modeling using Modeller 10.1, using crystallography data from Protein Data Bank 7bw1 as the template (Sali and Blundell, 1993).

### 2.5 Statistical Analysis

Analytical data were evaluated considering at least three independent replicates using the GraphPad Prism software (Version 9.0; GraphPad Software, Inc.; San Diego, CA, United States). Statistical analyses were conducted using one-way ANOVA. Differences were considered significant at *p* < 0.05.

## 3 Results

### 3.1 Clinical Characterization

#### 3.1.1 Subject 1

Family 1 included two brothers, 20 and 18 years old, with ambiguous external genitalia. None of the relatives has a reported history of gynecomastia. According to the parents, their marriages were not consanguineous. Other relevant antecedents of the current condition were denied by the parents.

The participant was an 8-year-old infant, the condition began from birth with the presence of two nodulations, one on each side of the vaginal tubercle, and the presence of the thickening of the clitoris, without treatment. The family visited the hospital, where hormonal studies were conducted in the Human Reproduction Biology laboratory, integrating the diagnosis of male with DSD. The child presented a short phallus (length 1.5 cm), penoscrotal hypospadias with pseudovagina, bilateral inguinal hernia (bilateral testes) within the labioscrotal folds, external genitalia with hypoplastic vaginal introitus, and thickening of the vaginal folds and labia majora, and hypertrophy of the clitoris. Unfused scrotal bag folds were observed, divided by a perineoscrotal cleft, which when introducing an infant feeding tube leads to the bladder. In the scrotal bags, some small nodulations are palpated (one per bag), which is compatible with a gonad of approximately 1 × 1 × 1.2 cm. In the genitalia, no hyperpigmentation was observed.

#### 3.1.2 Subject 2

Patient 2 was a 50-year-old woman raised as a girl. Pelvic ultrasound and magnetic resonance imaging revealed no Müllerian structures. The patient underwent gonadectomy.

The two participants were assigned to the female sex at birth. A summary and comparison of the clinical and biochemical parameters of the two subjects is presented in Table 2. The ratio of testosterone to DHT (T:DHT) after hCG stimulation was elevated in the two affected subjects, which had a T:DHT ratio >10 (Table 2).

**TABLE 2:** Biochemical characteristics of affected subjects.

	LH (IU/L)	FSH (IU/L)	Testosterone (nmol/L)	DHT (nmol/L)	Ratio T/DHT
Family 1					
Subject 1	0.70	0.099	7.6	0.4	18.5
Family 2					
Subject 2	8.0	12.1	22.3	0.5	43.0

Note. Abbreviations: LH, luteinizing hormone; FSH, follicle-stimulating hormone; DHT, dihydrotestosterone.

### 3.2 Molecular Analysis of SRD5A2 Gene

**FIGURE 2:**
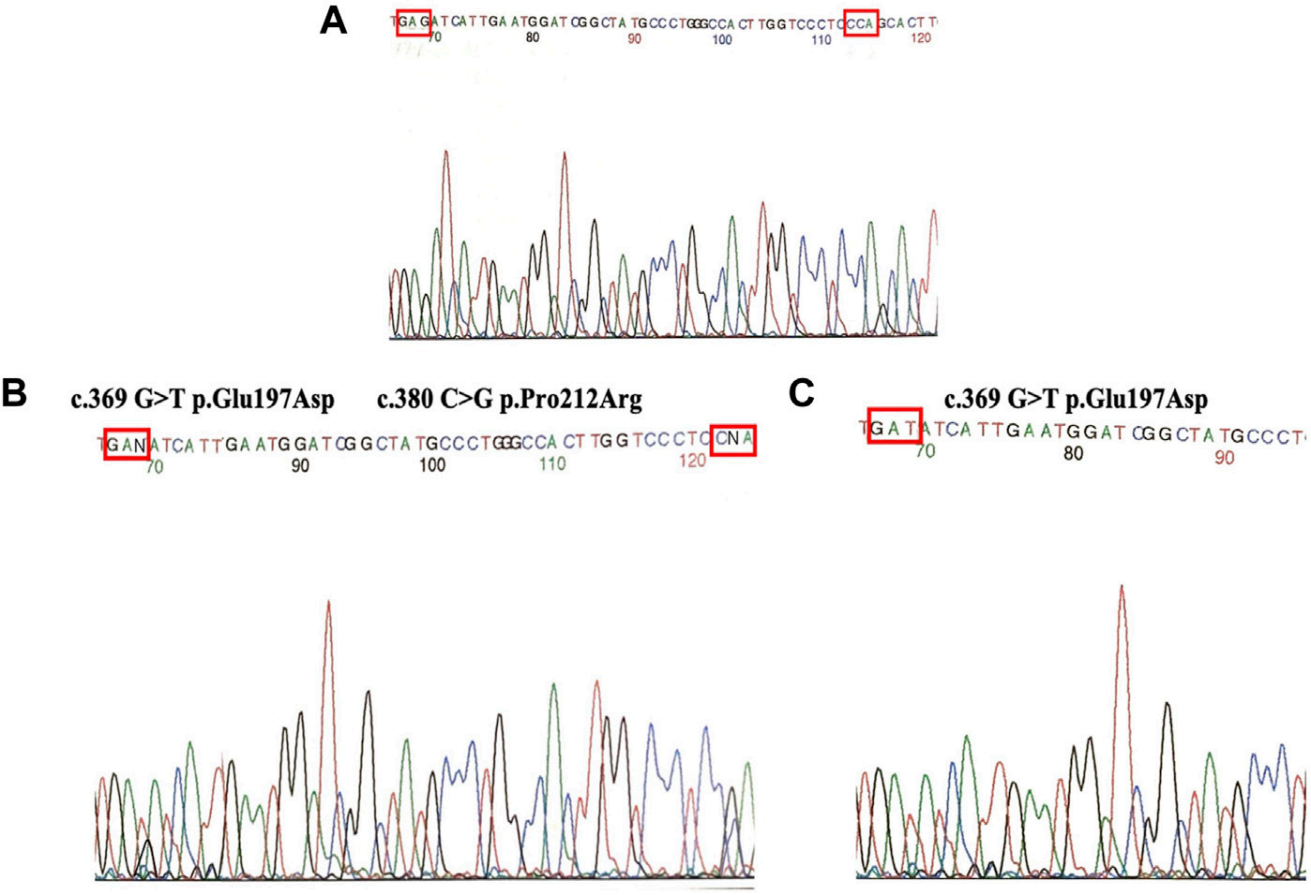
**(A)** Direct sequencing of wild type. **(B)** Heterozygous (Subject 1) for two mutations (p.Glu197Asp and p.Pro212Arg). **(C)** Homozygous individuals (Subject 2) for mutation P.Glu197Asp.

Sequencing analysis of exon 4 in both families showed the presence of two genetic variants p.Glu197Asp and p.Pro212Arg (Figure 2B) when compared with wild type (Figure 2A). Subject 1 was heterozygous for genetic variants. Both parents were carriers; the father had a change in codon p.Glu197Asp, and the mother had a change in codon p.Pro212Arg.

Notably, subject 2 was homozygous for the genetic variant p.Glu197Asp. In each family, the parents were carriers of the corresponding genetic variant (Figure 2C).

### 3.3 5a-Reductase Biological Activity

**FIGURE 3:**
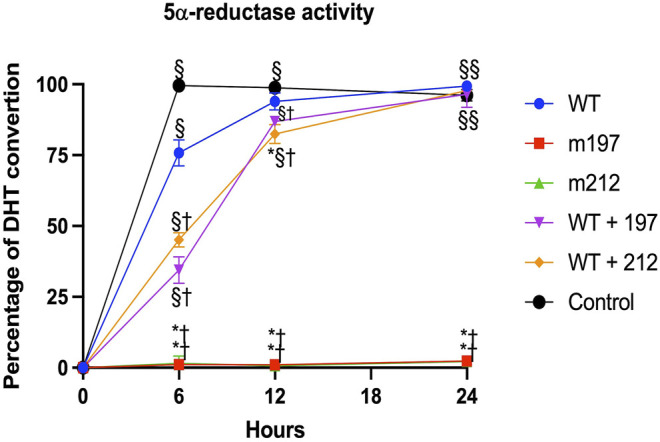
Steriod 5α-reductase 2 activity expressed in HEK293 cells. Saturation curves of mutants were determined by measuring the conversion of T [1, 2, 6, 7-3H(N)] to DHT with increasing concentrations of unlabeled T (0.125–8.0 mmil/L). All experiments were performed in triplicate. *p* < 0.05 *vs. WT; † *vs.* Control; § *vs.* m197, m212.

For determining the effect of these genetic variants on 67 5α‐R2 activity, cDNA constructs encoding normal, and mutant enzymes were transfected into cultured HEK-293 cells. A representative curve showing the activity of these *SRD5A2* mutants to convert T into DHT is shown in Figure 3.

The site-directed mutagenesis technique showed that the presence of the p.Glu197Asp or p.Pro212Arg variants led to a total loss of enzyme activity. Notably, the p.Glu197Asp variant has been described in heterozygous and homozygous forms in unrelated families [19, 20]. This study described a family affected by a homozygous p.Glu197Asp variant, which resulted in a total loss of 5α‐R2 activity and, consequently, abnormal genital development in these patients. The p.Glu197Asp and p.Pro212Arg variants carried in a compound heterozygote form completely lack the enzyme activity (Figure 3).

### 3.4 Prediction of Functional Effects of Mutant SRD5A2 and Structure Modeling

**FIGURE 4:**
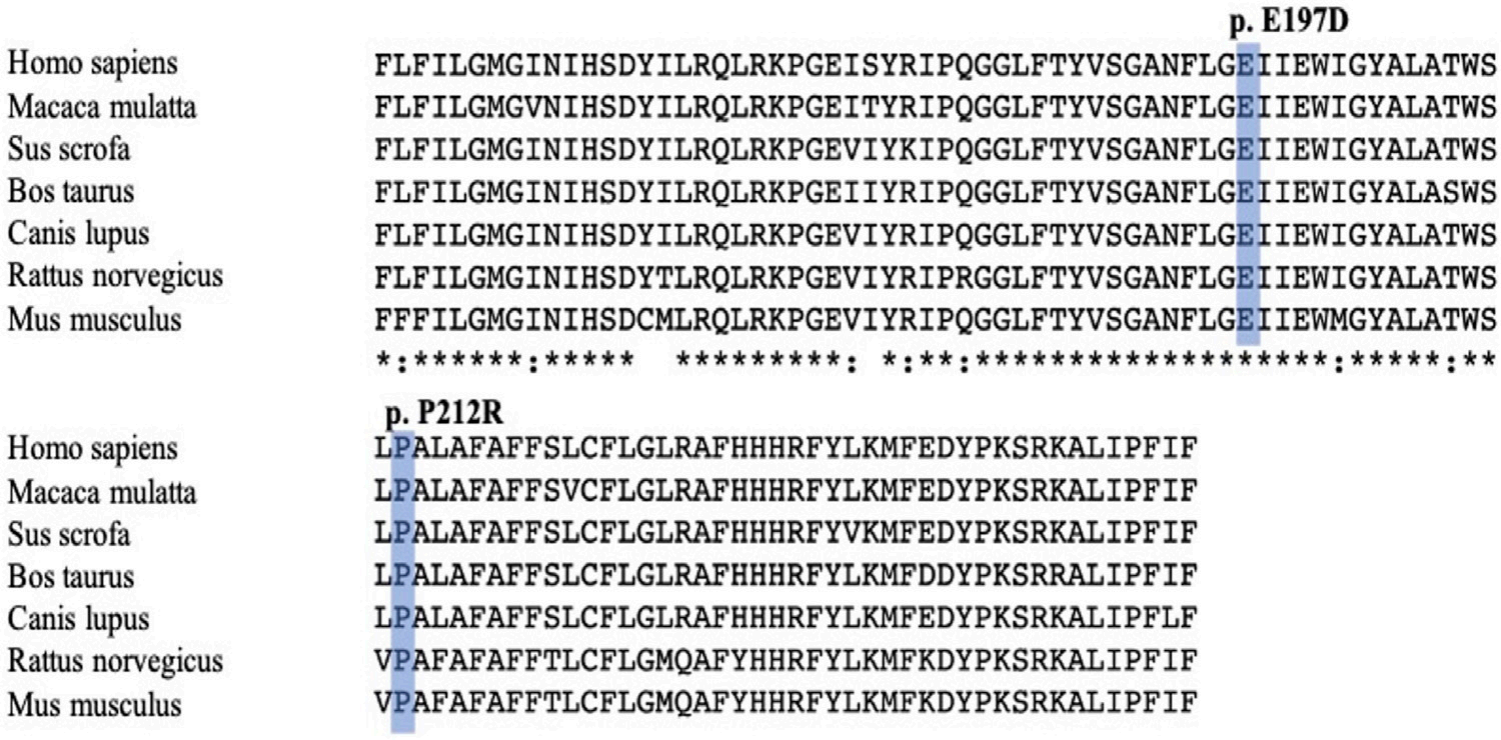
Comparison between multiple sequence alignment of human steriod 5α-reductase type 2 and other sequence proteins vertebrate reductases. Mutations reported in this study are shaded blue. Alignment was performed with ClustalW program (http://clustalw.genome.jp/).

**FIGURE 5:**
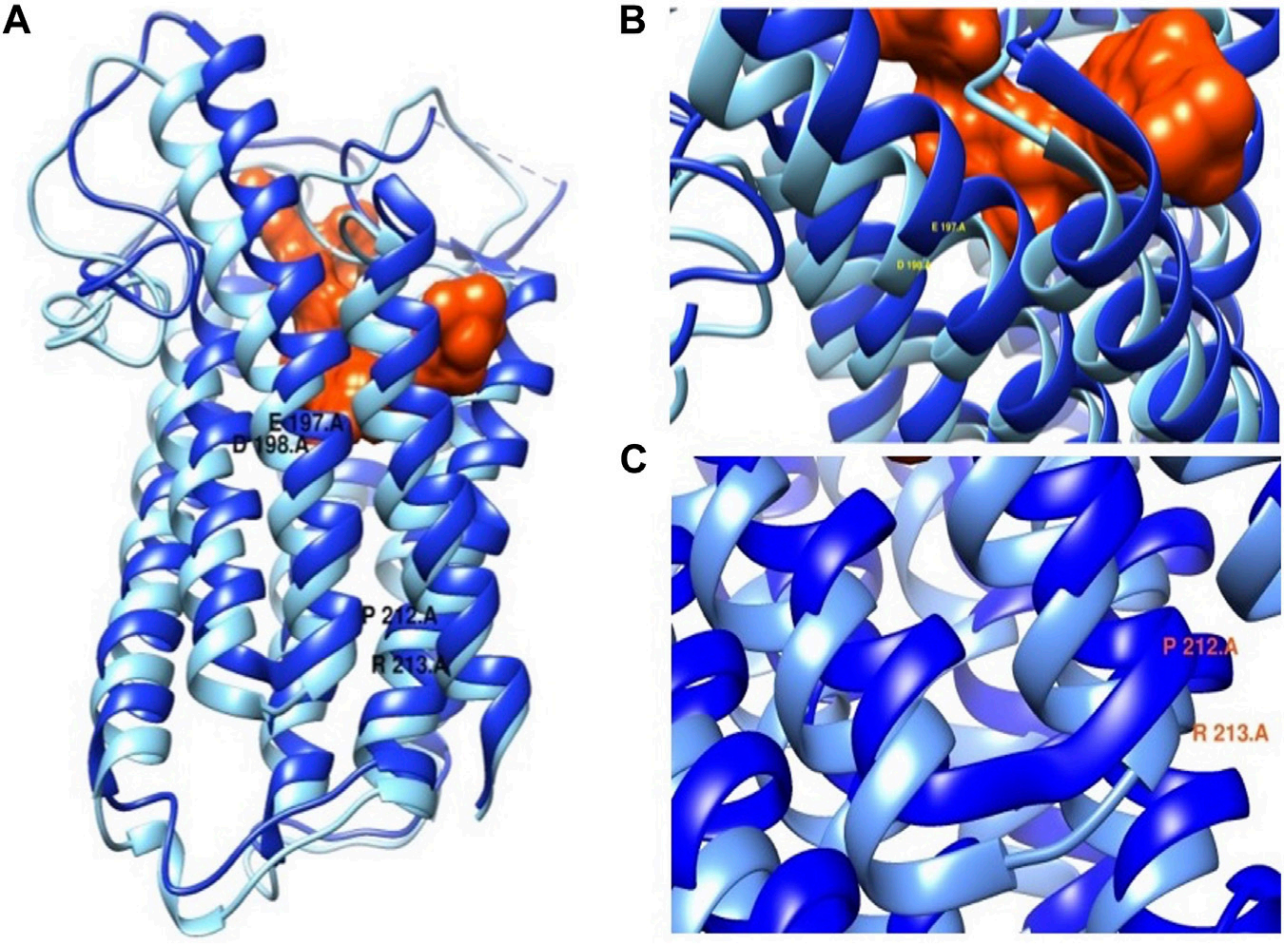
**(A)** SRD5A2 protein structure. **(B)** Mutation p.Glu197Asp close-up. **(C)** Mutation p.Pro212Arg close-up. In dark blue wild protein and in light blue mutated protein. The protein is in complex with finasteride orange.

Most functional predictions were deleterious, probably damaging or affecting the protein (Table 3). Both variants were in conserved regions (Figure 4). Structural modeling showed that residue 197 is found in transmembrane helix 6 and interacts with the nicotinamide of NADPH. By contrast, residue 212 is found in the transmembrane helix 7 near the amino terminal that faces the endoplasmic reticulum lumen (Figure 5).

**TABLE 3:** Functional predictions of amino acid changes detected in 5a-R2 in families studied.

Amino acid change	Polyphen-2 (Score near to 1.0)	PROVEAN (Score < −2.5)	SIFT (Score < 0.05)	CADD (Score)	MUpro (Score < 0)
p.Glu197Asp	Probably Damaging (0.934)	Deleterius (−2.906)	Affect protein function (0.00)	Likely benign (24)	Decrease stability (−0.788)
p.Pro212Arg	Probably Damaging (0.988)	Deleterius (−4.997)	Tolerated (0.32)	Likely benign (24)	Decrease stability (−0.393)

Note. PolyPhen2, polymorphism phenotyping; PROVEAN, Protein Variation Effect Analyzer; SIFT, sorting intolerant from tolerant amino acid substitutions; MUpro, (predictions of protein stability changes upon genetic variations.

The Exome Aggregation Consortium (ExAc) reported a frequency of 3.5 × 10^−4^ for the genetic variant p.Glu197Asp (rs121434253), and the genome Aggregation Database (gnomAD) reported a frequency of 9 × 10^−5^. By contrast, the ExAc reported a frequency of 3.3 × 10^−5^ for the genetic variant p.Pro212Arg (rs121434252), and the gnomAD reported a frequency of 1.2 × 10^−5^. The two pathogenic variants are rare and have been observed only in the Mexican population.

## 4 Discussion

In the present study, we show two cases of male individuals with an enzymatic defect of 5α-R2 manifested by ambiguous external genitalia at birth. The families of the subjects resided in a different geographic area. Each family had three affected siblings, all of whom had 46,XY DSD. The two studied subjects had karyotype 46,XY and were affected by 5α-RD2.

The *SRD5A2* gene from the parents and subjects was screened. The variants p.Glu197Asp and p.Pro212Arg were identified in conserved regions, suggesting that these positions are crucial for enzyme activity. The causative role of these genetic variants was confirmed using site-directed mutagenesis.

Additionally, the molecular diagnosis of 5α-RD2 in two Mexican children with 46,XY DSD was established. Two genetic variants of *SRD5A2* were detected in each subject. The inheritance pattern corresponds to an autosomal recessive disorder. The directed mutagenesis study showed that p.Glu197Asp and p.Pro212Arg are pathogenic and carried in a compound heterozygote form lead to a total loss of enzymatic activity and, consequently, abnormal genitalia development in the patients. In this study, for the first time, it was demonstrated that enzymatic activity with the genetic variant p.Glu197Asp is reduced; however, when this variant is present homozygously, the activity of the enzyme is completely lost (Figure 4).

These findings are consistent with those of Vilchis et al. (2010). Moreover, *in vitro* experiments using site-directed mutagenesis revealed that the p.Pro212Arg and p.Glu197Asp mutant enzymes showed complete loss of catalytic activity of 5α-R2 (Figure 3). These two pathogenic variants cause amino acid replacement. In the variant p.Glu197Asp, the substitution is a polar amino acid by another polar amino acid, and the structures of Glu and Asp are similar, differing by a methyl group; hence, the first hypothesis is that this change would not affect the function of the protein. Nonetheless, residue 197 is near the NADPH binding, which may destabilize NADPH binding. Regarding p. Pro212Arg, the substitution is a change in a cyclic side chain amino acid (apolar) by a positively charged amino acid. The pathogenic variant led to structural destabilizing replacement (Figure 5) (Han et al., 2021). Therefore, the p.Pro212Arg/p.Glu197Asp pathogenic variants produce enzymes with impaired activity and are unable to catalyze the synthesis of DHT during embryogenesis, explaining the possible phenotypic alterations in patients with 46, XY DSD. Studies have demonstrated that these pathogenic variants are highly recurrent in the Mexican population; hence, authors have suggested that they derive from a common ancestor by a founder gene effect responsible for the distribution of the defective enzyme activity of 5α-R2 (Chávez et al., 2000; Vilchis et al., 2000; Hackel et al., 2005; Skordis et al., 2005; Baldinotti et al., 2008; Vilchis et al., 2010). Notably, early studies have suggested the existence of genetic variant hot spots in this DNA region (Hackel et al., 2005; Vilchis et al., 2010). Both variants of the *SRD5A2* gene were located within codons 197–230. Wilson et al. proved that variation in this region caused complete inactivation of 5α-R2. This region contains a sequence of 21 amino acids (residues 206–226), which corresponds to one of the four transmembrane domains of the 5α-R2 enzyme (http://ca.expasy.org/uniprot/P31213) (Wilson et al., 1993). The impact of variants at this site indicates the importance of exon 4 of *SRD5A2*, which encodes part of transmembrane domains as a fundamental region for the correct functioning of 5α-R2 (Vilchis et al., 2008).

Although many variants of the *SRD5A2* gene have been reported and described worldwide, the genotype–phenotype correlation among patients has not been definitively revealed, even for individual carrying the same variant. We found that patients of Mexican origin with the variants p.Pro212Arg and p.Glu197Asp presented with perineoscrotal hypospadias, microphallus, cryptorchidism, and a female sex of rearing, demonstrating a potential genotype–phenotype correlation for these particular variants, which results in no enzymatic activity when present in a compound heterozygote form or homozygously.

## 5 Conclusion

In this study, we identified two pathogenic variants in the *SRD5A2* gene in two patients with 46,XY from two Mexican families. The subjects were heterozygous and homozygous for *SRD5A2* variants. The genetic variants p.Pro212Arg and p.Glu197Asp were located within exon 4 of the region considered functional domain for NADPH binding. The site-directed mutagenesis study molecular analysis of these families indicated that the affected patients had a rare form of 5α-RD2, caused by impaired synthesis of DHT. The results from this study allowed us to conclude that the cause of these patients having the classical form of 5α-RD2 was the genetic variants in exon 4 of the *SRD5A2* gene.

Finally, molecular diagnosis of 46,XY DSD is an advantageous tool for the diagnosis and subclassification of steroid 5α-RD2. Although the external appearance of genitalia continues to be the choice of assigned sex, additional factors influence sex development from birth until puberty. Gender assignment in children with DSD is a matter for a multidisciplinary team. Thus, improving the understanding of the underlying pathophysiology involving social and medical personnel that could provide the correct, opportune diagnosis is necessary.

## Data Availability

The datasets for this article are not publicly available due to concerns regarding participant/patient anonymity. Requests to access the datasets should be directed to the corresponding author.

## References

[B1] Acién P. Acién M. (2020). Disorders of Sex Development: Classification, Review, and Impact on Fertility. J. Clin. Med. 9, 3555. 10.3390/jcm9113555 PMC7694247 33158283

[B2] Ahmed S. F. Achermann J. C. Arlt W. Balen A. Conway G. Edwards Z. (2016). Society for Endocrinology UK Guidance on the Initial Evaluation of an Infant or an Adolescent with a Suspected Disorder of Sex Development (Revised 2015). Clin. Endocrinol. (Oxf) 84, 771–788. 10.1111/cen.12857 26270788 PMC4855619

[B3] Andersson S. Berman D. M. Jenkins E. P. Russell D. W. (1991). Deletion of Steroid 5α-Reductase 2 Gene in Male Pseudohermaphroditism. Nature 354, 159–161. 10.1038/354159a0 1944596 PMC4451825

[B4] Avendaño A. Paradisi I. Cammarata-Scalisi F. Callea M. (2018). 5-α-Reductase Type 2 Deficiency: Is There a Genotype-Phenotype Correlation? A Review. Hormones 17, 197–204. 10.1007/s42000-018-0013-9 29858846

[B5] Baldinotti F. Majore S. Fogli A. Marrocco G. Ghirri P. Vuerich M. (2008). Molecular Characterization of 6 Unrelated Italian Patients with 5 -Reductase Type 2 Deficiency. J. Androl. 29, 20–28. 10.2164/jandrol.107.002592 17609295

[B6] Chávez B. Valdez E. Vilchis F. (2000). Uniparental Disomy in Steroid 5 -Reductase 2 Deficiency. J. Clin. Endocrinol. Metab. 85, 3147–3150. 10.1210/jc.85.9.3147 10999800

[B7] Dallob A. L. Sadick N, S. Unger W. Lipert S. Geissler L. A. Gregoire S. L. (1994). The Effect of Finasteride, a 5 Alpha-Reductase Inhibitor, on Scalp Skin Testosterone and Dihydrotestosterone Concentrations in Patients with Male Pattern Baldness. J. Clin. Endocrinol. Metab. 79, 703–706. 10.1210/jc.79.3.703 8077349

[B8] Fan L. Song Y. Polak M. Li L. Ren X. Zhang B. (2020). Clinical Characteristics and Genotype-Phenotype Correlations of 130 Chinese Children in a High-Homogeneity Single-center Cohort with 5α-Reductase 2 Deficiency. Mol. Genet. Genomic Med. 8, e1431. 10.1002/mgg3.1431 32713132 PMC7549558

[B9] Grant D. B. Laurance B. M. Atherden S. M. Ryness J. (1976). HCG Stimulation Test in Children with Abnormal Sexual Development. Arch. Dis. Child. 51, 596–601. 10.1136/adc.51.8.596 9030 PMC1546104

[B10] Hackel C. Oliveira L. E. C. Ferraz L. F. C. Tonini M. M. O. Silva D. N. Toralles M. B. (2005). New Mutations, Hotspots, and Founder Effects in Brazilian Patients with Steroid 5α-Reductase Deficiency Type 2. J. Mol. Med. 83, 569–576. 10.1007/s00109-005-0651-7 15770495

[B11] Han Y. Zhuang Q. Sun B. Lv W. Wang S. Xiao Q. (2021). Crystal Structure of Steroid Reductase SRD5A Reveals Conserved Steroid Reduction Mechanism. Nat. Commun. 12, 449. 10.1038/s41467-020-20675-2 33469028 PMC7815742

[B12] Hughes I. A. Houk C. Ahmed S. F. Lee P. A. (2006). Consensus Statement on Management of Intersex Disorders. J. Pediatr. Urol. 2, 148–162. 10.1016/j.jpurol.2006.03.004 18947601

[B13] Imperato-McGinley J. Guerrero L. Gautier T. Peterson R. E. (1974). Steroid 5α-Reductase Deficiency in Man: An Inherited Form of Male Pseudohermaphroditism. Science 186, 1213–1215. 10.1126/science.186.4170.1213 4432067

[B14] Kang H.-J. Imperato-McGinley J. Zhu Y.-S. Rosenwaks Z. (2014). The Effect of 5α-Reductase-2 Deficiency on Human Fertility. Fertil. Sterility 101, 310–316. 10.1016/j.fertnstert.2013.11.128 PMC4031759 24412121

[B15] Labrie F. Sugimoto Y. Luu-The V. Simard J. Lachance Y. Bachvarov D. (1992). Structure of Human Type II 5 Alpha-Reductase Gene. Endocrinology 131, 1571–1573. 10.1210/endo.131.3.1505484 1505484

[B16] Lee P. A. Houk C. P. Ahmed S. F. Hughes I. A. (2006). Consensus Statement on Management of Intersex Disorders. Pediatrics 118, e488–e500. 10.1542/peds.2006-0738 16882788

[B17] Maimoun L. Philibert P. Cammas B. Audran F. Bouchard P. Fenichel P. (2011). Phenotypical, Biological, and Molecular Heterogeneity of 5α-Reductase Deficiency: An Extensive International Experience of 55 Patients. J. Clin. Endocrinol. Metab. 96, 296–307. 10.1210/jc.2010-1024 21147889

[B18] Miller S. A. Dykes D. D. Polesky H. F. (1988). A Simple Salting Out Procedure for Extracting DNA from Human Nucleated Cells. Nucl. Acids Res. 16, 1215. 10.1093/nar/16.3.1215 3344216 PMC334765

[B19] Özbey H. Etker S. (2013). Disorders of Sexual Development in a Cultural Context. Arab J. Urol. 11, 33–39. 10.1016/j.aju.2012.12.003 26579242 PMC4442941

[B20] Sali A. Blundell T. L. (1993). Comparative Protein Modelling by Satisfaction of Spatial Restraints. J. Mol. Biol. 234, 779–815. 10.1006/jmbi.1993.1626 8254673

[B21] Skordis N. Patsalis P. C. Bacopoulou I. Sismani C. Sultan C. Lumbroso S. (2005). 5α-Reductase 2 Gene Mutations in Three Unrelated Patients of Greek Cypriot Origin: Identification of an Ancestral Founder Effect. J. Pediatr. Endocrinol. Metab. 18, 241–246. 10.1515/jpem.2005.18.3.241 15813602

[B22] Vilchis F. Méndez J. P. Canto P. Lieberman E. Chávez B. (2000). Identification of Missense Mutations in the SRD5A2 Gene from Patients with Steroid 5α-Reductase 2 Deficiency. Clin. Endocrinol. 52, 383–387. 10.1046/j.1365-2265.2000.00941.x 10718838

[B23] Vilchis F. Ramos L. Mendez J. P. Benavides S. Canto P. Chavez B. (2010). Molecular Analysis of the SRD5A2 in 46,XY Subjects with Incomplete Virilization: The P212R Substitution of the Steroid 5 -Reductase 2 May Constitute an Ancestral Founder Mutation in Mexican Patients. J. Androl. 31, 358–364. 10.2164/jandrol.109.009407 20019388

[B24] Vilchis F. Valdez E. Ramos L. García R. Gómez R. Chávez B. (2008). Novel Compound Heterozygous Mutations in the SRD5A2 Gene from 46,XY Infants with Ambiguous External Genitalia. J. Hum. Genet. 53, 401–406. 10.1007/s10038-008-0274-2 18350250

[B25] Walsh P. C. Madden J. D. Harrod M. J. Goldstein J. L. MacDonald P. C. Wilson J. D. (1974). Familial Incomplete Male Pseudohermaphroditism, Type 2. N. Engl. J. Med. 291, 944–949. 10.1056/nejm197410312911806 4413434

[B26] Wigley W. C. Prihoda J. S. Mowszowicz I. Mendonca B. B. New M. I. Wilson J. D. (1994). Natural Mutagenesis Study of the Human Steroid 5.alpha.-Reductase 2 Isoenzyme. Biochemistry 33, 1265–1270. 10.1021/bi00171a029 8110760

[B27] Wilson J. D. Griffin J. E. Russell D. W. (1993). Steroid 5α-Reductase 2 Deficiency. Endocr. Rev. 14, 577–593. 10.1210/edrv-14-5-577 8262007

[B28] Wisniewski A. B. Batista R. L. Costa E. M. F. Finlayson C. Sircili M. H. P. Dénes F. T. (2019). Management of 46,XY Differences/Disorders of Sex Development (DSD) throughout Life. Endocr. Rev. 40, 1547–1572. 10.1210/er.2019-00049 31365064

